# Atosiban-Associated Non-cardiogenic Pulmonary Edema in a Dichorionic Diamniotic Twin Pregnancy: A Case Report

**DOI:** 10.7759/cureus.113838

**Published:** 2026-08-03

**Authors:** Simon Maigre, Virginie Meurant

**Affiliations:** 1 Intensive Care Unit, CHU Tivoli, La Louvière, BEL; 2 Emergency Medicine, CHU Tivoli, La Louvière, BEL

**Keywords:** atosiban, noncardiogenic pulmonary edema, preterm labor and obstetric care, tocolysis, twin pregnancy

## Abstract

Non-cardiogenic pulmonary edema is a rare but potentially life-threatening complication of tocolytic therapy. While commonly associated with beta-agonists and calcium channel blockers, its occurrence with atosiban remains poorly documented. To date, only a small number of cases have been reported in the literature.

We report the case of a 21-year-old primigravida with a dichorionic diamniotic twin pregnancy who developed acute hypoxemic respiratory failure during atosiban therapy for threatened preterm labor. A comprehensive diagnostic workup excluded cardiogenic, thromboembolic, and infectious causes. Imaging findings were consistent with pulmonary edema. Rapid clinical improvement following discontinuation of atosiban strongly supported a drug-related etiology.

This case highlights a rare but clinically relevant adverse effect of atosiban and emphasizes the importance of early recognition, particularly in high-risk patients.

## Introduction

Preterm labor remains a major cause of neonatal morbidity and mortality worldwide and affects approximately 5%-18% of pregnancies, depending on the population studied [1]. Tocolytic therapy is frequently administered to delay delivery for at least 48 hours, thereby allowing completion of antenatal corticosteroid therapy and maternal transfer to specialized perinatal centers when necessary [2].

Several classes of tocolytic agents are currently available, including β2-adrenergic agonists, calcium channel blockers, nonsteroidal anti-inflammatory drugs, and oxytocin receptor antagonists. Among these, atosiban, a competitive antagonist of oxytocin and vasopressin receptors, is widely used in Europe because of its favorable maternal safety profile and high uterine selectivity [1,3]. Large randomized controlled trials have demonstrated significantly fewer maternal cardiovascular adverse events with atosiban than with β2-adrenergic agonists, supporting its reputation as one of the safest available tocolytic agents [3].

Acute pulmonary edema is an uncommon but potentially life-threatening complication of pregnancy. In the obstetric setting, pulmonary edema may result from both cardiogenic and non-cardiogenic mechanisms and has been associated with several conditions, including preeclampsia, multiple gestation, excessive fluid administration, antenatal corticosteroid therapy, and tocolytic treatment [4,5-8]. Tocolysis-related pulmonary edema has been most frequently described with β2-adrenergic agonists and, to a lesser extent, calcium channel blockers [2,4,5].

In contrast, pulmonary edema associated with atosiban remains exceptionally rare. A recent systematic review by Yang et al. identified only seven published cases of atosiban-associated acute pulmonary edema, most of which occurred in women with multiple pregnancies and concomitant exposure to antenatal corticosteroids [9]. Despite the widespread use of atosiban in clinical practice, the pathophysiological mechanisms underlying this adverse event remain poorly understood, and awareness among clinicians remains limited.

We report a case of suspected atosiban-associated non-cardiogenic pulmonary edema in a primigravida with a dichorionic diamniotic twin pregnancy and discuss this observation in the context of the currently available literature.

## Case presentation

A 21-year-old primigravida was admitted at 24 weeks and four days of gestation for threatened preterm labor. The pregnancy was a dichorionic diamniotic twin gestation resulting from in vitro fertilization with the transfer of two frozen embryos. Her past medical and surgical history was notable for splenectomy following traumatic splenic injury during childhood, previous surgery for endometriosis, self-reported asthma without formal pulmonary evaluation or maintenance therapy, and a documented penicillin allergy of an unspecified nature.

Regarding the current pregnancy, the patient remained normotensive throughout gestation, and repeated urine dipstick testing showed no evidence of proteinuria. She had gestational diabetes mellitus managed with dietary measures and metformin, without the need for insulin therapy.

On admission (Day 0), she presented with regular uterine contractions and vaginal bleeding. Vital signs were stable, with a blood pressure of 120/60 mmHg, a heart rate of 88 beats/min, an oxygen saturation of 98% on room air, and a body temperature of 36.6°C. Clinical examination confirmed preterm premature rupture of membranes. Fetal monitoring was reassuring for both fetuses. Initial laboratory investigations revealed mild anemia associated with neutrophilic leukocytosis and low inflammatory markers (Table 1).

**Table 1:** Laboratory investigations during hospitalization ^*^Arterial blood gas analysis was performed while the patient was receiving 3 L/min of supplemental oxygen via nasal cannula. PaO_2_ , partial arterial pressure of oxygen; PaCO_2_, partial arterial pressure on carbon dioxide

Parameter	Normal value	Value, day 1	Value, day 2
Hemoglobin (g/dL)	12.0-16.0	9.4	9.5
White blood cell count (/mm^3^)	4000-10000	20230	23300
Neutrophils (/mm^3^)	1500-7500	18900	20900
C-reactive protein (mg/L)	<5	4.3	2.0
PaO_2 _(mmHg)	80-100	-	67^*^
PaCO_2 _(mmHg)	35-45	-	27^*^

Management included initiation of atosiban with a 6.75 mg intravenous bolus followed by a continuous infusion at 300 µg/min for the first three hours and 100 µg/min thereafter. Magnesium sulfate was administered for fetal neuroprotection. Because of preterm premature rupture of membranes, empirical antibiotic therapy with amoxicillin 2 g every six hours was started. Fetal lung maturation was promoted with betamethasone 12 mg on Day 0 and a second dose on Day 1.

Approximately 48 hours after initiation of atosiban therapy, the patient developed progressive dyspnea and hypoxemia requiring supplemental oxygen therapy. An extensive diagnostic workup was performed, including repeat laboratory testing, viral polymerase chain reaction (PCR) assays, arterial blood gas analysis, chest radiography, transthoracic echocardiography, and pulmonary perfusion scintigraphy.

Repeat laboratory testing demonstrated worsening neutrophilic leukocytosis with persistently low inflammatory markers. Arterial blood gas analysis confirmed hypoxemic respiratory failure associated with hypocapnia (Table 1). PCR testing for influenza viruses and SARS-CoV-2 was negative.

**Figure 1:**
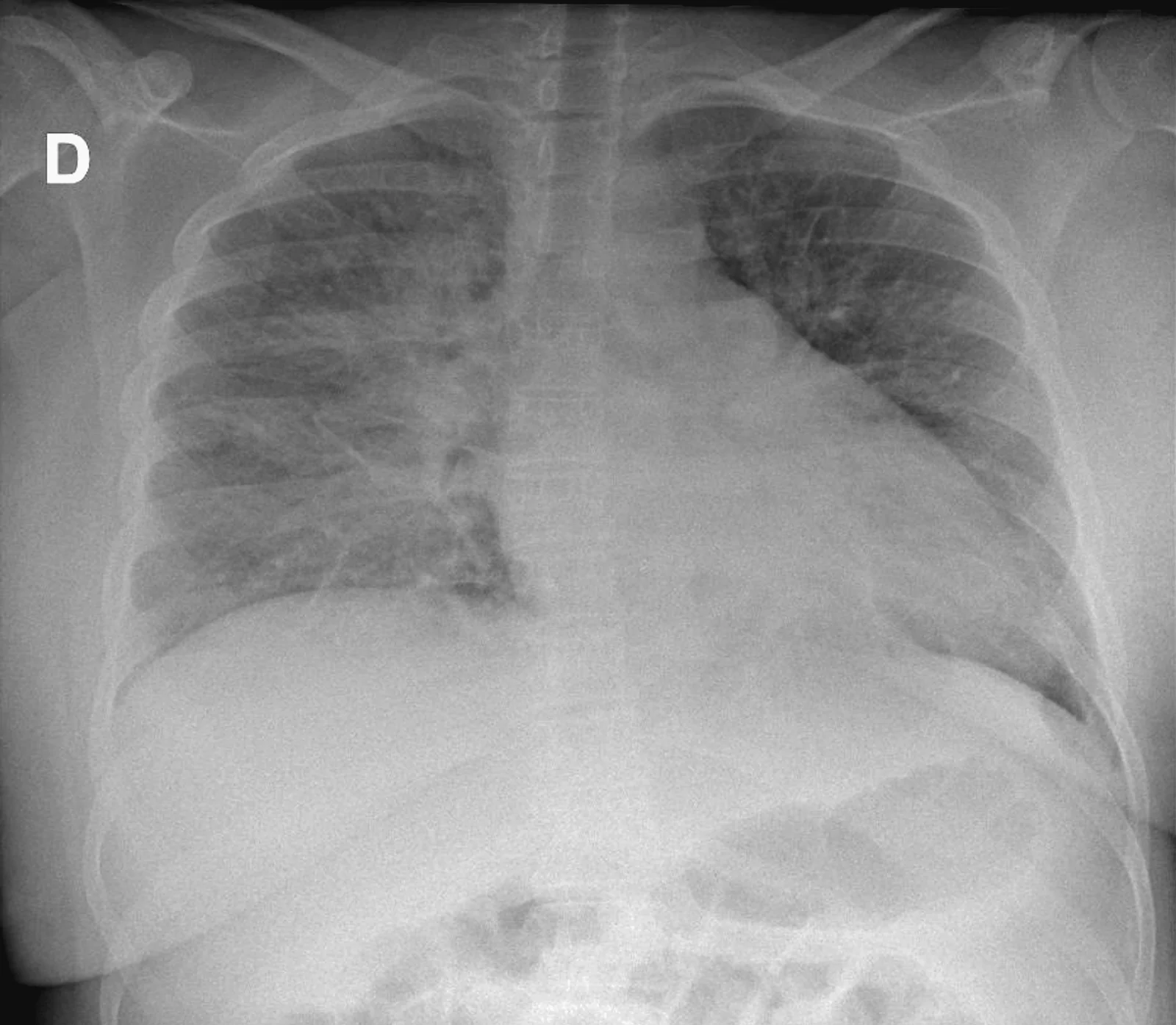
Chest X-ray Chest radiograph obtained on Day 2, showing diffuse bilateral alveolar-interstitial infiltrates predominantly involving the perihilar and lower lung fields, consistent with non-cardiogenic pulmonary edema.

Chest radiography demonstrated bilateral diffuse alveolar-interstitial infiltrates (Figure 1). Transthoracic echocardiography showed preserved left ventricular systolic function (LVEF, 60%) without evidence of elevated filling pressures. Right ventricular function was normal, with no indirect signs of pulmonary hypertension. The inferior vena cava was small and collapsed during spontaneous inspiration, suggesting the absence of significant volume overload. Pulmonary perfusion scintigraphy showed no evidence of pulmonary embolism.

On Day 3, owing to worsening respiratory failure with increasing oxygen requirements up to an fraction of inspired oxygen (FiO₂) of 60%, the patient was transferred to the intensive care unit. Given the absence of cardiac dysfunction and the temporal relationship to tocolytic therapy, a diagnosis of atosiban-associated non-cardiogenic pulmonary edema was considered the most likely etiology. Management consisted of immediate discontinuation of atosiban, administration of loop diuretics, and respiratory support with alternating continuous positive airway pressure (CPAP) and high-flow nasal oxygen therapy. A marked improvement in oxygenation was observed within hours of discontinuation of atosiban. By Day 4, less than 24 hours after treatment withdrawal, oxygen requirements had decreased to 3-5 L/min via low-flow nasal cannula. Later that same day, the patient underwent an uncomplicated vaginal delivery. Postpartum recovery was characterized by rapid respiratory improvement, allowing complete discontinuation of oxygen therapy and return to room air within a short period.

## Discussion

We report a case of suspected atosiban-associated non-cardiogenic pulmonary edema in a young woman with a dichorionic diamniotic twin pregnancy treated for threatened preterm labor. Acute pulmonary edema is a recognized complication of tocolytic therapy and has been extensively described with β2-adrenergic agonists and, to a lesser extent, calcium channel blockers [3-5]. In contrast, pulmonary edema associated with atosiban remains exceptionally rare and is likely underrecognized.

A recent systematic review by Yang et al. identified seven published cases of atosiban-associated acute pulmonary edema, including the index case reported by the authors [6]. Most patients were nulliparous (85.7%) and carrying multiple pregnancies (71.4%). All had received antenatal corticosteroids, and the median interval between atosiban initiation and symptom onset was approximately 40 hours [6]. Interestingly, the characteristics of our patient closely mirrored those identified in the systematic review, including nulliparity, twin gestation, exposure to antenatal corticosteroids, and symptom onset approximately 48 hours after initiation of atosiban therapy, further supporting the plausibility of a causal association.

Several confounding factors should nevertheless be acknowledged. Our patient received magnesium sulfate, betamethasone, and amoxicillin in addition to atosiban. Furthermore, twin gestation itself constitutes a recognized risk factor for pulmonary edema during pregnancy because of the greater hemodynamic and metabolic adaptations required [7,8].

Pregnancy induces profound cardiovascular and respiratory physiological changes that may predispose susceptible patients to pulmonary edema. Plasma volume expansion exceeds the increase in red blood cell mass, resulting in physiological hemodilution and reduced plasma oncotic pressure. Cardiac output increases through both elevated stroke volume and heart rate, while systemic and pulmonary vascular resistance decreases. These adaptations create a physiological state in which relatively minor additional insults may precipitate pulmonary fluid accumulation [8,9].

The pathophysiology of pulmonary edema associated with β2-agonists and calcium channel blockers is relatively well established [3,4]. Reflex tachycardia may shorten diastolic filling time and increase cardiac filling pressures, while corticosteroids may contribute to fluid retention through mineralocorticoid effects. Twin pregnancy further amplifies these physiological changes.

The mechanism underlying atosiban-associated pulmonary edema remains uncertain. Atosiban is a competitive antagonist of both oxytocin and vasopressin receptors and is considered the most uterus-selective tocolytic agent currently available [1,2]. Beyond its uterine effects, experimental studies suggest that oxytocin and vasopressin may exert protective actions on the cardiopulmonary system [10,11]. Therefore, inhibition of these pathways could theoretically contribute to increased pulmonary vascular permeability and the development of non-cardiogenic pulmonary edema. However, direct evidence supporting this hypothesis remains limited.

The temporal association between atosiban administration, the onset of respiratory symptoms, the exclusion of alternative etiologies, including pulmonary embolism and cardiogenic pulmonary edema, and the rapid clinical improvement following drug withdrawal strongly supports a causal relationship in the present case.

Large randomized controlled trials have consistently demonstrated a favorable cardiovascular safety profile for atosiban compared with β2-agonists [2]. In a multinational randomized trial involving 735 women treated with either atosiban or β2-agonists, only one case of pulmonary edema was reported in the atosiban group, occurring after previous exposure to β2-agonist therapy [2]. Cardiovascular adverse events occurred in only 8.3% of patients receiving atosiban compared with 81.2% of those receiving β2-agonists, highlighting the rarity of severe cardiopulmonary complications associated with atosiban.

Although uncommon, clinicians should remain aware of this potentially serious adverse event, particularly in patients with recognized risk factors such as multiple gestation, antenatal corticosteroid exposure, prolonged tocolysis, or concomitant administration of other medications associated with fluid retention.

## Conclusions

Non-cardiogenic pulmonary edema is a rare but potentially life-threatening complication of atosiban therapy. Although the overall safety profile of atosiban remains favorable compared with that of other tocolytic agents, clinicians should consider this diagnosis in pregnant women who develop unexplained respiratory deterioration during treatment.

The present case adds to the limited body of evidence suggesting a possible association between atosiban and acute pulmonary edema, particularly in the setting of multiple gestation and concomitant obstetric therapies. Prompt recognition, discontinuation of the suspected agent, and supportive respiratory management appear to be associated with rapid clinical recovery. Further studies are required to clarify the causal relationship and elucidate the underlying pathophysiological mechanisms responsible for this uncommon adverse event.

## References

[1] Romero R, Sibai BM, Sanchez-Ramos L (2000). An oxytocin receptor antagonist (atosiban) in the treatment of preterm labor: a randomized, double-blind, placebo-controlled trial with tocolytic rescue. Am J Obstet Gynecol.

[2] Haas DM, Caldwell DM, Kirkpatrick P, McIntosh JJ, Welton NJ (2012). Tocolytic therapy for preterm delivery: systematic review and network meta-analysis. Br Med J.

[3] Worldwide Atosiban versus Beta-Agonists Study Group (2001). Effectiveness and safety of the oxytocin antagonist atosiban versus beta-adrenergic agonists in the treatment of preterm labour. BJOG.

[4] Lamont RF (2000). The pathophysiology of pulmonary oedema with the use of beta-agonists. BJOG.

[5] Abbas OM, Nassar AH, Kanj NA, Usta IM (2006). Acute pulmonary edema during tocolytic therapy with nifedipine. Am J Obstet Gynecol.

[6] Dennis AT, Solnordal CB (2012). Acute pulmonary oedema in pregnant women. Anaesthesia.

[7] Norwitz ER, Edusa V, Park JS (2005). Maternal physiology and complications of multiple pregnancy. Semin Perinatol.

[8] Soma-Pillay P, Nelson-Piercy C, Tolppanen H, Mebazaa A (2016). Physiological changes in pregnancy. Cardiovasc J Afr.

[9] Yang Z, Wu W, Yu Y, Liu H (2023). Atosiban-induced acute pulmonary edema: a rare but severe complication of tocolysis. Heliyon.

[10] Gutkowska J, Jankowski M (2012). Oxytocin revisited: its role in cardiovascular regulation. J Neuroendocrinol.

[11] Jankowski M, Broderick TL, Gutkowska J (2020). The role of oxytocin in cardiovascular protection. Front Psychol.

